# Microbiological Biodiversity of Regional Cow, Goat and Ewe Milk Cheeses Produced in Poland and Antibiotic Resistance of Lactic Acid Bacteria Isolated from Them

**DOI:** 10.3390/ani13010168

**Published:** 2022-12-31

**Authors:** Beata Nalepa, Lidia Hanna Markiewicz

**Affiliations:** 1 Department of Food and Industrial Microbiology, Faculty of Food Technology, University of Warmia and Mazury, Pl. Cieszynski 1, 10-726 Olsztyn, Poland; 2 Department of Immunology and Food Microbiology, Institute of Animal Reproduction and Food Research of the Polish Academy of Sciences, Tuwima 10, 10-748 Olsztyn, Poland

**Keywords:** oscypek, redykołka, tvarog, bryndza, regional cheese, lactic acid bacteria, antibiotic resistance, food safety

## Abstract

**Simple Summary:**

Traditional and regional cheeses are becoming more and more popular among consumers. Usually, they are produced in small dairy plants which are characterized by a unique microbiota of the processing area. The unique microbiota determines both the microbial quality and safety of the final products. Another issue related to the microbiological safety of food products is the presence of antibiotic resistance (AR) in microbiota, which is a potential health issue for humans. The AR was mainly investigated in pathogenic bacteria as a direct risk for effective antimicrobial therapy. However, the AR phenomenon is also present in desired food bacteria participating in the production of dairy products. These bacteria can be a reservoir of antibiotic resistance genes (ARG) in the environment, therefore monitoring AR in LABs seems to be an urgent need for ensuring the safety of food. In this work, we investigated the microbial diversity of ripened and unripened cheeses produced from cow, ewe, and goat milk in Poland, and identified LAB typical for cheeses and microbiota characteristic of the investigated types of cheese. Moreover, we assessed the phenotypic AR and the presence of ARG in lactic acid bacteria.

**Abstract:**

(1) Unique sensory values of traditional and regional dairy products made them more and more popular among consumers. Lactic acid bacteria naturally occurring in these products can express antibiotic resistance and be a reservoir of antibiotic resistance genes (ARG) in the environment. The aim of the study was to characterize the microbial diversity of twenty regional cheeses produced from non-pasteurized cow, goat and ewe milk, and investigate the phenotypic and genotypic antibiotic resistance (AR) of lactic acid bacteria isolated from these products. (2) Conventional microbiological methods were applied for the enumeration of lactic acid bacteria (lactobacilli and lactococci) and their isolation, and for the enumeration of *Enterococcus*, *Staphylococcus*, *Enterobacteriaceae* and spores. The disc diffusion method was applied for phenotypic AR. The PCR-based methods were used for strain identification, microbiological diversity of cheeses (PCR-DGGE), and for AR gene detection. (3) Among 79 LAB isolates the most frequent species were *L. plantarum* (*n* = 18), *Leuc. lactis* (*n* = 17), *Lc. lactis* (*n* = 11), *Leuc. mesenteroides* (*n* = 9) and *L. pentosus* (*n* = 8). Additionally, by using the PCR-DGGE method, DNA of *L. casei* was found in nine products. Lactobacilli (5.63–8.46 log cfu/g) and lactococci (6.15–8.41 log cfu/g) predominated over *Enterococcus* (max. 4.89 log cfu/g), *Staphylococcus* (max. 4.18 log cfu/g), and *Enterobacteriaceae* (mostly up to 4.88 log cfu/g). Analysis of phenotypic resistance to tetracycline (30 µg), erythromycin (15 µg), and chloramphenicol (30 µg) showed that 29% of LAB isolates were resistant to one antibiotic, 8%—to two, and 12%—to all tested antibiotics. Antibiotic resistance genes (AGR) for tetracycline (*tet(M)*, *tet(L)*, *tet(W)*), erythromycin (*erm(B)*) and chloramphenicol (*cat-TC*) were detected in 30 (38%), 29 (36.7%) and 33 (43.4%) LAB isolates, respectively. Among 31 LAB isolates phenotypically susceptible to all tested antibiotics, only 5 (16%) had no ARGs. (4) The results obtained in our work shed light on the potential threat posed by the widespread presence of ARGs in LAB present in regional cheeses.

## 1. Introduction

Regional and traditional milk products are becoming more and more popular among consumers due to their nutritional values and unique sensory characteristics [1]. The tradition of producing rennet cheeses (both ripened and unripened) in Poland applies to the most popular cow milk and goat and ewe milk. The most popular traditional Polish cheeses are made in Podhale and are based on ewe, cow or goat milk, or a mixture of cow and ewe milk [2], and these are “oscypek” which together with “redykołka” (mini variety of oscypek) and “bryndza podhalańska” (soft rennet cheese) are registered under the European Protected Designation of Origin (PDO) geographical indication [3]. Other popular traditionally produced cheeses in Poland are bundz and curd fresh cheese “twaróg” (tvarog) which are on the List of Traditional Products in the Malopolska Region [3]. Tvarog and other regionally produced rennet cheeses are manufactured from cow’s milk in all regions of Poland, whereas bundz is made of ewe milk.

Regional and artisanal dairy products made in small dairy plants are characterized by microorganisms representing local, often unique microbiota whose composition and metabolic activity determine the exceptional sensory characteristics of cheeses. The final microbial composition of a product is a result of the microbiota of raw milk, hygienic procedures applied in the plant, and technological processes such as milk pasteurization, fermentation, and ripening used for the manufacturing of the product [4,5,6]. The presence of lactic acid bacteria is necessary for the manufacture of the product, and also limits the development of undesirable microflora that can spoil the product or pose a risk to the health of consumers.

An important aspect of food product safety is the presence of antibiotic resistance (AR) in both desirable and undesirable bacteria present in the specific product. Spreading antibiotic resistance is one of the major health threats worldwide, therefore monitoring the antibiotic resistance in bacteria consumed with food is one of the ways for predicting the threat to human health. Antibiotic resistance in bacteria can be an inherent and intrinsic trait encoded by genes located on chromosomal DNA, it is not transferable to other bacteria. Acquired antibiotic resistance is coded by genes located on mobile genetic elements (plasmids or transposons), and it can be transferred to other bacteria via horizontal gene transfer, therefore contributing to the increased risk of AR spreading in the environment [7].

AR has been widely investigated in pathogens present in raw milk and milk products, such as *Salmonella*, *Staphylococcus aureus*, *Listeria monocytogenes*, or *Escherichia coli* [8,9,10,11] which is obviously linked with their direct danger to human health and with consequences for effective treatment in case of infection. Research of antibiotic resistance in LABs naturally present in milk products is equally important because they, as an essential element of the products and the production environment, can act as an AR reservoir from which AR genes (AGRs) can be transferred to other bacteria. Despite that, data on the phenomenon of AR in dairy LAB concern mainly industrial LAB strains [12], and probiotics [7] whereas the area of regional milk products including cheese still needs to be explored. To the best of our knowledge, the present work investigates for the first time both phenotypic and genotypic antibiotic resistance in lactic acid bacteria present in regional cheeses produced in Poland.

The studies aimed to characterize the microbial diversity of regional cheeses produced from cow, goat and ewe milk and investigate the antibiotic resistance of lactic acid bacteria isolated from these products. Microbial diversity was investigated using standard microbiological methods and the previously established, culture-independent PCR-DGGE method [13]. The safety of cheeses was evaluated based on the presence of antibiotic resistance (phenotypic and genetic background) in isolated lactic acid bacteria.

## 2. Materials and Methods

### 2.1. Cheese Samples

Twenty cheese samples were purchased on local markets from regional producers in northeastern (Warmia and Mazury) and southern (Podhale) Poland. Only natural cheeses (non-smoked, without additives such as herbs or fruits) made from non-pasteurized were included in the study (Table 1) and these were: 15 samples of ripening cheeses (ewe-cow milk “oscypek”—7, ewe milk “oscypek”—1, goat milk oscypek-like cheese—1, redykołka—3, cow milk cheese—2, goat milk cheese—1) and five samples of unripened cheeses (ewe milk fresh white cheese “bundz”—1, ewe milk white cheese “bryndza”—1, cow milk fresh white cheese “twaróg”—2, goat milk fresh white cheese (twaróg)—1). All products were produced in the Spring (April or May).

### 2.2. Microbial Diversity Determined by PCR-DGGE

#### 2.2.1. DNA Isolation and Polymerase Chain Reaction (PCR)

The DNA was isolated directly from cheese samples with the Genomic Mini AX FOOD Kit (A@A Biotechnology, Gdańsk, Poland) in accordance with the manufacturer’s instructions. The PCR reactions were carried out in the MJ Mini Gradient Thermal Cycler (Bio-Rad, Warszawa, Poland) with primers U968-GC (5′-CGCCCGGGGCGCGCCCCGGGCGGGGCGGGGGCACGGGGGGAACGCGAAGAACCT TAC-3′) and L1401-r (5′-CGGTGTGTACAAGACCC-3′) [14] which amplify the V6-V8 region of the 16S rRNA coding gene. The reaction mixture (25 µL) consisted of a 1× reaction PCR buffer (20 mmol/L Tris-HCl, pH 8.4, 50 mmol/L KCl, 3 mmol/L MgCl_2_, 50 μmol/L deoxyribonucleotides (dNTPs), 5 pmol/L of each primer), 1.25 U *Taq* polymerase (Thermo Fisher Scientific, Warszawa, Poland) and about 20 ng of the DNA template. In each PCR run a non-template control and positive control of amplification with DNA from one of the reference strains (Online Resources Supplementary Table S1) was included. The PCR profile was as follows: initial denaturation at 94 °C for 5 min, followed by 35 cycles of denaturation at 94 °C for 30 s, annealing at 56 °C for 30 s and extension at 68 °C for 40 s. The final extension was carried out at 68 °C for 7 min [14].

#### 2.2.2. Denaturing Gradient Gel Electrophoresis (DGGE)

The PCR products (~450 bp) were analyzed by denaturing gradient gel electrophoresis (DGGE) with urea and formamide (Sigma, Poznań, Poland) as denaturing agents as described previously [13]. In short, electrophoresis was carried out in 8% polyacrylamide gel (acrylamide:bis-acrylamide, 37.5:1) (Sigma, Poznań, Poland) with the denaturing gradient ranging from 35% to 57.5%. Electrophoresis was conducted in 0.5× Tris-acetate-EDTA (Sigma, Poznań, Poland) buffer at 60 °C and 85 V for 16 h [14] in the DCode Universal Mutation System (Bio-Rad, Warszawa, Poland). Gels were stained in SybrGreen I (1:10,000) (Sigma) solution for 15 min and documented under UV light in G-Box (Syngen, Wrocław, Poland). On each gel, previously developed markers [13] were run to enable the identification of bacterial species. Bacterial species used for designing the DGGE makers set are listed in Online Resources Supplementary Table S1.

### 2.3. Enumeration of Selected Bacterial Groups

Cheese samples (10 g) were homogenized in buffered peptone water (Merck, Warszawa, Poland), serially diluted, plated on appropriate agar media (all supplied by Merck), and incubated in conditions suitable for the target bacterial groups (Table 2). The counts of bacterial genera: *Lactobacillus, Lactococcus, Enterococcus, Staphylococcus, Clostridium, Bacillus* and *Enterobacteriaceae* were determined. Spores of *Clostridium* spp. and *Bacillus* spp. were determined in samples after heat treatment (80 °C, 10 min).

### 2.4. Isolation and Identification of Lactic Acid Bacteria from Cheese Samples

#### 2.4.1. Isolation of Lactic Acid Bacteria

Cheese samples (10 g) were homogenized in 90 mL of buffered peptone water (Merck) and plated on MRS or M17 agar (Merck) and incubated 24–48 h at 30 or 37°C. Two to 10 different colonies with typical LAB morphology were selected and grown in liquid MRS and M17 media (30 or 37 °C/24–48 h). Next, 1 mL of each culture was transferred to an Eppendorf tube and centrifuged (10,000 rpm, 10 min, room temperature (RT)). The pellet was resuspended in TE buffer and DNA was isolated using the Genomic Mini AX FOOD Kit (A@A Biotechnology, Gdańsk, Poland) in accordance with the manufacturer’s instructions. Isolated DNA was spectrophotometrically quantified and stored at −20 °C.

#### 2.4.2. Identification of Lactic Acid Bacteria Isolates

Isolated LABs were identified using a PCR method with primers published previously [15,16,17,18,19,20,21,22,23,24,25] and conditions listed in Supplementary Table S1 (Online Resources Supplementary Table S2). The reaction mixture (25 µL) consisted of 1× reaction PCR buffer (20 mM/L Tris-HCl, pH 8.4, 50 mM/L KCl, 2 mM/L MgCl_2_, 200 μM/L deoxyribonucleotides (dNTPs), 5 pmol/L of each primer), 1 U *Taq* polymerase (Thermo Fisher Scientific, Life Technologies, Warszawa, Poland) and 10−40 ng of the DNA template. In each PCR run a non-template control and positive control of amplification with DNA isolated from an appropriate reference strain (Online Resources Supplementary Table S1) was included. Amplification was performed in a Thermal Cycler (PTC-200, MJ Research, Reno, NV, USA). The temperature profile was as follows: initial denaturation at 94 °C for 5 min, followed by 35 cycles of denaturation at 94 °C for 30 s, annealing temperature given in Supplementary Table S1 for 30 s and extension at 68 or 72 °C for 40 s. The final extension was carried out at 68 or 72 °C for 7 min. The length of PCR products was confirmed by electrophoresis in a 1.5% agarose gel (Promega, Poland) in 1× TBE buffer (pH 8.3) stained with ethidium bromide. Gels were photographed under UV light and documented using the G-BOX system (Syngen, Wrocław, Poland).

### 2.5. Testing of Phenotypic Antibiotic Resistance

Antimicrobial susceptibility was determined using the disc diffusion method. Three antibiotics were tested: tetracycline (TE, 30 μg), erythromycin (E, 15 μg), and chloramphenicol (C, 30 μg) since resistance to them is one of the most frequently reported among dairy LABs [26,27]. Cartridges with commercially prepared paper discs containing the appropriate antibiotic dosage were purchased from Oxoid (Argenta, Poznań, Poland). Disk diffusion assays were performed on Mueller–Hinton Agar (Merck). Overnight cultures of LAB isolates were spotted on the surface of the Mueller–Hinton agar. Antibiotic discs were then placed on the plates and incubated at 30 or 37 °C. Zone diameters were recorded after a 24-h incubation period. *Escherichia coli* ATCC 25922, *Enterococcus faecalis* ATTC 29212 and *Staphylococcus aureus* ATTC 25923 were used as the reference resistant strains. There are no CLSI (https://clsi.org, accessed on 21 November 2022) or EUCAST (https://www.eucast.org/eucastguidancedocuments, accessed on 21 November 2022) criteria for lactic acid bacteria, therefore the strains were considered resistant when the zone of inhibition of growth was ≤18 mm [28].

### 2.6. Determination of Genotypic Antibiotic Resistance

The presence of the resistance genes to tetracycline (*tet*M, *tet*W, *tet*L), erythromycin (*erm(B*)) and chloramphenicol (*cat*-*TC*) was determined by PCR method using specific primers [29,30,31,32,33] (Online Resource Supplementary Table S3). A non-template control and positive control of amplification with DNA isolated from an appropriate reference strain (Online Resources Supplementary Table S1) were included. DNA isolation of LAB strains and PCR mixture was described above. PCR-based detection of the genes *tet*(M), *tet*(L), *tet*(W), *erm(B)* and *cat*-*TC* was performed under the following conditions: 94 °C for 3 min; 94 °C for 30 s, annealing temperature (Supplementary Table S2) for 30 s and 72 °C for 30 s (35 cycles); and 72 °C for 5 min. Amplification products were detected by electrophoresis in a 1.5% agarose gel (Promega, Madison, WI, USA), stained with ethidium bromide and documented using the G-BOX system (Syngen).

## 3. Results

### 3.1. Microbial Diversity and Quality Determined by PCR-DGGE

The number of species and their diversity determined with the use of PCR-DGGE depended on the type of product analyzed and the region of its origin (Table 3). The lowest number of species was detected in unripened cheese type produced in Podhale—in bryndza (three species: *Lc. lactis*, *L. casei* and *C. freundii*) and in bundz cheese (four species: *Leuc. mesenteroides*, *L. brevis*, *E. aerogenes* and *L. monocytogenes*), and in oscypek cheese (from three species in Os7 to 7 species in Os2, Table 3). In products from the Warmia and Mazury, at least 8 species of bacteria in ripening goat cheese Sg2 and up to 16 species in ripening Sg1 goat cheese was found (Table 3).

In all oscypek cheeses (Os1-Os8, Og1) the DNA of lactic acid bacteria was present, and the most frequently found species were *Lc. lactis*, *Leuc. mesenteroides* and *L. casei* (in six, five and four oscypek samples, respectively) whereas the least frequent were *L. plantarum* and *L. brevis* (in three and in one sample, respectively) (Table 3). All oscypek cheeses contained DNA of one up to three species among fecal bacteria: *E. coli*, *C. freundii*, *E. aerogenes*, *E. cloacae* and *E. faecalis*. In oscypek cheeses, the undesirable species were also found: *S. aureus* (five samples), *Cl. butyricum* (two), *Cl. tyrobutyricum* (two) and *Propionibacterium jensenii* (one), and *L. monocytogenes* (one).

In redykołek-type cheeses the diversity of bacterial species was higher—lactic acid bacteria were represented by *Lc. lactis*, *Leuc. mesenteroides*, *L. casei*, *L. fermentum*, and *L. acidophilus* in all three redykołek samples, whereas *L. brevis* and *L. helveticus* were found in two and one samples, respectively. The fecal bacterial species *E. aerogenes* and *C. freundii* were present in three and one redykołek cheese samples, respectively. Other bacterial species found in this cheese type were: *Pr. jensenii* (three samples), *S. aureus* (three), *Cl. tyrobutyricum* (one), and *L. monocytogenes* (one) (Table 3).

Cheeses produced in the Warmia and Mazury were characterized by higher microbiological diversity than those produced in Podhale. Lactic acid bacteria found in those cheeses were *Leuc*. *mesenteroides* (five samples), *L*. *plantarum* (five), *Lc. lactis* (four), *L. delbrueckii* (two), *L. casei* (one), *L. acidophilus* (one) and *L. helveticus* (one) (Table 3). Among other species considered desirable, the occurrence of *Str. thermophilus* in four samples and *Pr. jensenii* and *Pr. freudenreichii* in five samples were stated. Among the bacteria of fecal origin, the presence of *C. freundii*, *E. aerogenes*, *E. cloacae*, *E. coli*, and *E. faecalis* was found in five, four, four, two, and one cheese samples, respectively. Species considered undesirable especially in matured cheeses such as *Cl. tyrobutyricum*, *C. butyricum* and *C. perfringens* were found, respectively, in three, two, and one samples of the tested cheeses from the region of Warmia and Mazury. Other species whose DNA was present in those cheeses were *L. monocytogenes*, *S. aureus* and *S. xylosus* in six, five, and two samples, respectively (Table 3).

### 3.2. Microbiological Profiling of Cheeses

Among bacterial groups determined with classical methods, the numbers of streptococci belonging to the genus *Lactococcus*, and *Lactobacillus* were the highest and ranged from 6.15 (Os4) to 8.41 (Re1) log cfu/g, and from 5.63 (Os4) to 8.46 (Tr1) log cfu/g, respectively (Table 4). Fecal bacteria belonging to the *Enterobacteriaceae* family were determined in 14 (70%) of examined cheese samples in amounts greater than 100 cfu/g, and their level ranged from 2.00 (Os6) to 6.00 (Se1) log cfu/g. *E. coli* was present in nine samples at the level of 2.00 (Os6 and Sg2) to 3.70 (Ch1) log cfu /g.

Other bacteria of fecal origin *Enterococcus* spp. ranged from 3.04 (Tr1) to 5.75 (Se1) log cfu/g. *Staphylococcus* spp. was also present in cheeses in numbers ranging from 2.08 to 4.18 log cfu/g in Os4 and Re3, respectively. The presence of bacterial spores from the *Bacillus* and *Clostridium* genera was also determined and their number of more than 100 spores per 1 g was demonstrated in 6 samples and 7 samples, respectively. *Bacillus* abundance ranged from 2.11 to 2.90 log cfu/g, and *Clostridium*—from 2.20 to 3.00 log cfu/g (Table 4).

### 3.3. Isolation and Identification of LAB Strains

Seventy-nine strains of lactic acid bacteria were isolated from the tested regional cheeses (Online Resources Supplementary Table S4), half of them were Gram-positive cocci, and the other—were Gram-positive rods that formed colonies with a morphology typical for LAB. Their taxonomical identification revealed that 17 (22.4%) strains were *Leuconostoc lactis*, 10 (13.2%) strains were *Lactococcus lactis*, nine (11.8%) were *Leuconostoc mesenteroides*, and one strain (1.3%) belonged to *Lc. garviae* and *Enterococcus faecalis* (Online Resource Supplementary Table S4).

### 3.4. Phenotypic and Genotypic Resistance of Tested Strains

The analysis of the phenotypic sensitivity of isolated LABs to tetracycline, erythromycin, and chloramphenicol (Online Resources Supplementary Table S4), showed that 32 (40.5%) of them were sensitive to all tested antibiotics (Table 5). Among the 47 remaining strains, 36 (45.6%) showed resistance to tetracycline, 25 (31.6%) resistance to chloramphenicol, and 18 (22.8%) to erythromycin. The multidrug resistance observed in phenotypic tests was present in 9 (11%) strains resistant to three antibiotics, however, it should be noted that as many as 14 (18%) strains were resistant to two antibiotics.

The presence of tetracycline resistance genes: *tet(W)*, *tet(L)* and *tet(M)* was found in 3 (3.8%), 7 (9.2%) and 21 (26.6%) strains, respectively, while the erythromycin *erm(B)* and chloramphenicol *cat-TC* resistance genes were more common and were found in 29 (36.7%) and 33 (43.4%) isolated LABs (Table 5). In contrast, 13 isolates (16.5%) did not show any of the antibiotic resistance genes tested. Only 7 (8.9%) out of 79 isolates did not show both phenotypic and genotypic resistance to antibiotics (Table 5). As many as 24 strains (30.4%) showed multidrug genotypic resistance.

The comparison of the results of phenotypic and genotypic AR revealed that genotypic analysis confirmed 48% of the phenotypic resistance. What is interesting, among 31 LAB isolates phenotypically susceptible to all tested antibiotics, only 5 (16%) had no ARGs (Online Resources Supplementary Table S3). When comparing the AR in LABs isolated from products manufactured in the two regions, it occurred that among 42 Podhale-originated isolates 28 (67%) express the phenotypic AR and 38 (90%) had ARG. Of the 33 LABs originating from Warmia and Mazury region, 16 (48%) were phenotypically resistant to antibiotics, whereas 25 (76%) had ARG (Online Resources Supplementary Table S3).

## 4. Discussion

### 4.1. Microbial Diversity and Quality

In this study, we applied culture-dependent and culture-independent methods to evaluate the microbiological biodiversity and safety of traditional/regional and artisanal cheeses produced in Poland. We put attention to lactic acid bacteria, which are an indispensable component of fermented dairy products.

The results of the enumeration of bacteria obtained in our study were, in general, in line with previous reports. Analysis of the microbial composition showed that in all types of cheeses lactobacilli and lactococci predominated over other bacterial groups, reaching the level of 5.63–8.46 (lactobacilli) and 6.15–8.41 log cfu/g (lactococci) which was by several orders of magnitude higher compared to *Enterococcus* and *Staphylococcus* which did not exceed 4.89 and 4.18 log cfu/g, respectively. In previous studies, the levels of lactobacilli and lactococci in non-smoked oscypek cheese were approx. 8–9 log cfu/g and *Leuconostoc* about 7 log cfu/g [34], whereas in Slovak bryndza cheese the average counts of lactobacilli and lactococci were 6.6 × 10^8^ and 1.1 × 10^9^ cfu/g, respectively [35]. Data for tvarog, the typical Polish type of cow milk curd cheese, are scarce, however, it has been reported that cheeses with added LAB strains contained up to 6 log cfu/g lactobacilli [36]. In goat cheese produced in a small organic dairy plant, the LAB levels ranged from 7.82 to 8.11 log cfu/g [37]. The counts of *Enterococcus* and *Staphylococcus* reported by others were similar or higher compared to our results and, in general, ranging from 4.37 log cfu/g (*Enterococcus*) in goat cheese [37] up to 2 × 10^6^ cfu/g *Staphylococcus* in Slovak bryndza [35]. In our study, *Enterobacteriaceae* were present in 14 out of 20 tested cheese samples (the majority of cases were in the range of 2.28–4.88 log cfu/g). Reported enterobacteria levels in oscypek (average of 5.59 log cfu/g [34]), bryndza (9.0 × 10^3^–1.5 × 10^5^ cfu/g [35]) or goat cheese (4.36–6.66 log cfu/g [37]) were similar to our results.

The microbial diversity and quality of cheese result from the quality of the raw material and hygienic conditions during milk processing. What is more, the metabolic activity of microorganisms originating from both the raw material and the processing environment impacts the sensory quality and durability of cheese [13]. Additionally, the diversity of bacterial communities and the profile of sensory compounds strongly depend on the seasonality of cheese production [5,6]. The culture-independent methods detect the DNA of the microbiota of interest while do not discriminate between live and dead cells [38]. Therefore, the species composition revealed by using the PCR-DGGE method shows both species that are active in the tested cheese samples as well as species that were present in the raw material and in the products at any stage of the manufacturing process but not necessarily active in the final product. In our analysis, we used a primer pair universal for bacteria, which allowed us to characterize the DNA of the main bacterial taxa present in tested samples. A comparison of LAB species detected by PCR-DGGE (Table 3) and results of LAB isolates identification (Online Resources Supplementary Table S3) showed that in some cases (*e.g.,* sample Os1, Og1, Re2, Sg2) the obtained results were not consistent. Similar discrepancies in the LAB diversity obtained by culture-dependent and culture-independent identification were observed by others [38,39]. Taking into account the bias of PCR amplification (differences in the detectability threshold of different targeted species and the masking effect of the most abundant templates during PCR) [38] we can support the statement that the use of both approaches gives the most complete picture of the microbial composition that was and/or is currently active in the product.

Nevertheless, the obtained results are in general consistent with reports on the LAB species present in these types of dairy products. The LAB composition in oscypek cheeses is in line with the work by Alegria et al. [34] who also detected *Lc. lactis*, *Leuc. mesenteroides*, and *L. plantarum*. Species of non-enterococcal LAB isolated from Slovak ovine cheese and bryndza were identified as *L. casei*/*L. paracasei*, *L. plantarum*, *L. rhamnosus*, *L. helveticus*, *L. delbrueckii*, *L. fermentum*, *L. brevis*, *Lc. lactis*, *P. pentosaceus* and *P. acidilactici* [40]. Pangallo et al. [35] investigated the LAB composition of bryndza cheese with culture-independent methods (PCR-DGGE and cloning followed by sequencing) and reported the presence of DNA of *Lactococcus garvieae*, *Lactococcus lactis* subsp. cremoris, *Lactococcus lactis* subsp. *lactis*, *Mannheimia glucosida* (linked with mastitis in sheep), and *Streptococcus parauberis*. In tvarog cheese, *Lactococcus lactis* and *Leuconostoc mesenteroides* [41] are usually present which was confirmed in our studies by both culture-dependent and culture-independent methods.

In our study, the presence of genetic material of undesired bacteria of fecal origin and those that can cause spoilage of the final products (*Enterococcus faecalis*, *Enterobacter* spp., *Citrobacter freundii*, *Staphylococcus* spp., *Clostridium butyricum*, *Cl*. *tyrobutyricum*) [42,43,44] was detected. It should be noted that their presence was mostly consistent with the results of microbiota enumeration on agar media which revealed the presence of *Enterococcus* and *Staphylococcus* in all samples, *Enterobacteriaceae* in most of them and *Clostridium*—in only some of them (Table 4). The presence of undesirable microbiota that may contribute to food poisoning (*e.g., Listeria monocytogenes*, *Pseudomonas aeruginosa*, *Staphylococcus aureus*) was also stated, however not confirmed by culture methods. On the other hand, members of LAB but also *Enterococcus* and non-pathogenic *Staphylococcus* species are considered a natural microbiota of artisanal raw milk cheeses [45,46].

### 4.2. Antibiotic Resistance of LABs Isolated from Regional Cheeses

The occurrence of antibiotic resistance in bacteria derived from animal-origin products is an effect of the use of veterinary important antimicrobials in food-producing animals. Due to the direct threat to human and animal health and life, antibiotic resistance has been mainly investigated in pathogenic bacteria. In this study, we investigated the resistance of lactic acid bacteria to antibiotics that have great importance in human and animal medicine, as tetracycline belongs to class D, and erythromycin and chloramphenicol belong to class C of the antibiotics according to the classification of European Medicines Agency [47] what makes them potentially more frequently used compared to the antibiotics from category A and B. What is also of great importance, the ARGs for tetracycline, erythromycin and chloramphenicol are located on mobile genetic elements in LABs [48], therefore their presence may bring important consequences for spreading AR in pathogenic bacteria present in the environment.

The newest reports provided evidence of the presence of multidrug-resistant *Staphylococcus aureus* and *E. coli* in raw milk cheeses in America, Africa and Europe [8,9,46,49,50]. In Europe, coagulase-positive *S. aureus* strains were resistant, among others, to erythromycin (38.8% of tested strains isolated from raw milk cheeses in Romania) and tetracycline (22.4%) [46]. *Enterococcus faecalis* strains isolated from raw milk cheeses in Italy were phenotypically resistant to tetracycline (27.5% of the tested strains), rifampicin (7.5%), chloramphenicol (5%), and erythromycin (77%), whereas as many as 90% of the isolates had *tet(M)* gene and 30% had the *ermB* gene [50]. Regarding lactic acid bacteria, evidence for the presence of multidrug resistance in LAB isolated from commercially produced cheeses in China showed that the most frequent resistance was observed for streptomycin and sulfamethoxazole (100 and 91.7% of the isolates, respectively [12]). Up to now, data on the antibiotic resistance genes in LAB originating from raw milk dairy products are scarce. Morandi et al. [45] investigated LAB isolated from Italian raw milk curd and cheese and found that among 75 isolates none was phenotypically resistant to erythromycin, 19 (25.3%) were resistant to tetracycline, and as many as 47 (62.7%) and 39 (52%) were resistant to streptomycin and oxacillin, respectively. Despite the studies on regional cheeses made from raw milk are not numerous, some knowledge about the AR phenomenon in LABs can be gained from studies that aimed at the isolation of LABs from their natural environment and characteristics as potential probiotics. As can be expected, the pattern of susceptibility/resistance of LABs differs depending on the studied material, geographical location, and veterinary interventions to farm animals in the region. Ruiz-Monayo et al. [51] reported that all lactobacilli strains isolated from soft cheese in Portugal were found phenotypically susceptible or moderately susceptible to chloramphenicol, erythromycin, and tetracycline, as well as to penicillin G, ampicillin, gentamycin and clindamycin. This is in contradiction to our results showing that 57% of investigated LABs were resistant to at least one of three tested antibiotics: chloramphenicol (32% of tested strains), erythromycin (18%), and tetracycline (43%). On the other hand, lactobacilli isolated from traditional Turkish fermented dairy products were found resistant to erythromycin (10.8% of isolates), tetracycline (4.3%), gentamicin (28%), and ciprofloxacin (26%), whereas streptococci to vancomycin (40%), erythromycin (10%), chloramphenicol (10%), gentamicin (20%), and ciprofloxacin (30%) [27]. It should be noted here that the natural AR of LABs to vancomycin, nalidixic acid, kanamycin, polymyxin B and trimethoprim is present in most lactobacilli [48], and has been confirmed by Ruiz-Monayo et al. [51].

Although phenotypic testing for AR is the basis for the safety assessment of bacterial isolates, the assessment of potential risk connected to AR in strain or in a particular environment/product can be evaluated based on the presence of ARGs. The application of high-throughput sequencing technologies is being successfully applied for the characterization of microbiomes of traditional cheeses, as recently reported in a metagenomic study [52] where LABs present in Brazilian traditional cheeses have high levels of ARGs indicating the use of milk from animals undergoing antibiotic treatment. The genomic analysis of LAB isolates from bryndza cheese [53] revealed that only some of the tested LAB carried ARGs what excluded them from further application in food production.

Analysis of the microbial composition of fermented dairy products usually is associated with the functionality of starter and non-starter cultures and with a possible influence of the accompanying microbiota on sensory values and spoilage processes of the final product. In this study, we showed that antibiotic resistance is common in lactic acid bacteria isolated from different types of traditional and artisanal cheeses produced from raw (cow, ewe, or goat) milk, and provided data on the spread of antibiotic resistance in LABs in two regions of Poland. Further deepened studies on antibiotic resistance should be planned and performed on a higher number of samples that fulfil the requirements of the International Commission on Microbiological Specifications for Foods [54] regarding the acceptable risk in microbiological analysis. The future works should also include a wider range of antimicrobials to cover all antibiotic categories, and variables such as seasonality, regionality, and additional processes applied to cheeses (smoking, seasonings etc.) that may influence their microbiota.

Besides the direct impact of the microbiota composition on the sensory values of the final product and its microbiological safety (including the spreading of ARG), the bacteria present in dairy products may have a broader effect on consumers’ health. As recently investigated, proteins of lactic acid bacteria found in raw cow milk express immunomodulatory and antioxidant potential [55]. What is more, the enzymatic activity of bacteria present in fermented dairy products results in the formation of bioactive peptides derived from milk proteins, which can exert antimicrobial and ACE-inhibitory activity or promote mucin expression [56]. The abovementioned properties of bacterial proteins and enzymatic activity open new possibilities for a targeted selection of LAB and designing a new type of functional foods.

## 5. Conclusions

The obtained results showed the complementary of the applied methods for the evaluation of bacterial diversity of cheeses. By using classical methods, we enumerated lactic acid bacteria and undesired bacteria in cheeses and characterized the phenotypic antibiotic resistance in LABs isolated from them. With DNA-based methods, we analyzed the presence of genetic material of bacterial species in cheeses and the genetic background of antibiotic resistance in LAB isolates. The obtained results showed that lactic acid bacteria predominated in the tested cheeses and that most of them express phenotypic resistance to antibiotics. What seems to be more important, a vast majority of tested LAB isolates had antibiotic resistance genes, therefore the population of lactic acid bacteria found in regional cheeses can pose a potential source of ARGs in the environment.

## Figures and Tables

**Table 1:** Characteristics of studied cheeses.

Cheese Sample Symbol	Milk Species	Ripening	Origin	Regional Name
Os1	cow-ewe	yes	Podhale	oscypek
Os2	cow-ewe	yes	Podhale	oscypek
Os3	cow-ewe	yes	Podhale	oscypek
Os4	cow-ewe	yes	Podhale	oscypek
Os5	cow-ewe	yes	Podhale	oscypek
Os6	cow-ewe	yes	Podhale	oscypek
Os7	cow-ewe	yes	Podhale	oscypek
Os8	ewe	yes	Podhale	oscypek
Og1	goat	yes	Podhale	oscypek
Re1	cow-ewe	yes	Podhale	redykołka
Re2	cow-ewe	yes	Podhale	redykołka
Re3	cow-ewe	yes	Podhale	redykołka
Bu1	ewe	no	Podhale	bundz
Br1	ewe	no	Podhale	bryndza
Sg1	goat	yes	Warmia and Mazury	ser Koszałek
Sg2	goat	yes	Warmia and Mazury	Ser kozi
Se1	cow	yes	Warmia and Mazury	ser Baryłka
Ch1	goat	no	Warmia and Mazury	twarożek kozi
Tr1	cow	no	Warmia and Mazury	twaróg
Tr2	cow	no	Warmia and Mazury	twaróg

**Table 2:** Culture media and incubation conditions applied in the study.

Target Bacteria	Medium	Incubation Conditions
*Lactococcus*	M17 agar	30 °C, 48 h
*Lactobacillus*	De Man, Rogosa, Sharpe (MRS) agar	30 °C, 48 h, anaerobic *
*Enterobacteriaceae*	Violet Red Bile Lactose (VRBL) agar	37 °C, 24–48 h
*Enterococcus*	Stanetz-Bartley agar	37 °C, 48 h
*Staphylococcus*	Rabbit plasma fibrinogen (RPF) agar	37 °C, 48 h
*Clostridium*	Reinforced Clostridial Agar (RCM agar)	37 °C, 48 h, anaerobic
*Bacillus*	Nutrient agar	30 °C, 48 h

*Anaerobic conditions were obtained with the use of Anaerocult C bags (Merck, Warszawa, Poland).

**Table 3:** Microbial diversity of studied cheeses assessed by PCR-DGGE.

Cheese Samples	Species Detected by PCR-DGGE	
	Desirable/Beneficial	Undesirable/Pathogenic
Os1	*Lactococcus lactis*	*Clostridium butyricum*, *Citrobacter freundii*, *Enterobacter aerogenes*, *Escherichia coli*, *Staphylococcus aureus*
Os2	*Lactococcus lactis*, *Leuconostoc mesenteroides*, Lactiplantibacillus plantarum *	*Citrobacter freundii*, *Clostridium butyricum*, *Enterobacter aerogenes*, *Staphylococcus aureus*
Os3	*Lactococcus lactis*, *Propionibacterium jensenii*	*Enterococcus faecalis*, *Escherichia coli*, *Staphylococcus aureus*
Os4	*Lactococcus lactis*, *Leuconostoc mesenteroides*	*Escherichia coli*, *Clostridium tyrobutyricum*
Os5	Levilactobacillus brevis *, *Leuconostoc mesenteroides*	*Enterobacter cloacae*, *Clostridium tyrobutyricum*, *Staphylococcus aureus*
Os6	Lacticaseibacillus casei *, Lactiplantibacillus plantarum *	*Escherichia coli*, *Enterobacter cloacae*, *Listeria monocytogenes*, *Staphylococcus aureus*
Os7	*Leuconostoc mesenteroides*, *Lactobacillus casei*	*Escherichia coli*
Os8	*Lactococcus lactis*, Lactiplantibacillus plantarum *, Lacticaseibacillus casei *	*Citrobacter freundii*
Bu1	*Leuconostoc mesenteroides*, Levilactobacillus brevis *	*Enterobacter aerogenes*, *Listeria monocytogenes*
Br1	*Lactococcus lactis*, Lacticaseibacillus casei *	*Citrobacter freundii*
Og1	*Lactococcus lactis*, *Leuconostoc mesenteroides*, Lacticaseibacillus casei *	*Citrobacter freundii*
Re1	*Lactococcus lactis*, *Leuconostoc mesenteroides*, Lacticaseibacillus casei *, Limosilactobacillus fermentum *, *Lactobacillus acidophilus*, *Propionibacterium jensenii*	*Enterobacter aerogenes*, *Clostridium tyrobutyricum*, *Staphylococcus aureus*
Re2	*Lactococcus lactis*, *Leuconostoc mesenteroides*, *Levilactobacillus brevis*, Lacticaseibacillus casei*, Limosilactobacillus fermentum *, *Lactobacillus acidophilus*, *Propionibacterium jensenii*	*Citrobacter freundii*, *Enterobacter aerogenes*, *Staphylococcus aureus*
Re3	*Lactococcus lactis*, *Leuconostoc mesenteroides*, Lacticaseibacillus casei *, Levilactobacillus brevis *, *Lactobacillus helveticus*, Limosilactobacillus fermentum *, *Lactobacillus acidophilus*, *Propionibacterium jensenii*	*Citrobacter freundii*, *Enterobacter aerogenes*, *Listeria monocytogenes*, *Staphylococcus aureus*
Sg1	*Lactococcus lactis*, Lacticaseibacillus casei *, *Lactobacillus delbrueckii*, *Lactobacillus helveticus*, *Streptococcus thermophilus*, *Propionibacterium jensenii*, *Propionibacterium freudenreichii*	*Enterobacter cloacae*, *Enterococcus faecalis*, *Citrobacter freundii*, *Bacillus subtilis*, *Clostridium tyrobutyricum*, *Clostridium butyricum*, *Clostridium perfringens*, *Listeria monocytogenes*, *Staphylococcus aureus*
Sg2	*Leuconostoc mesenteroides*, Lactiplantibacillus plantarum *, *Streptococcus thermophilus*, *Propionibacterium jensenii*, *Propionibacterium freudenreichii*	*Enterobacter aerogenes*, *Bacillus subtilis*, *Listeria monocytogenes*
Se1	*Lactococcus lactis*, *Leuconostoc mesenteroides*, Lactiplantibacillus plantarum *, *Streptococcus thermophilus*, *Propionibacterium jensenii*, *Propionibacterium freudenreichii*	*Escherichia coli*, *Citrobacter freundii*, *Enterobacter aerogenes*, *Enterobacter cloacae*, *Listeria monocytogenes*, *Staphylococcus aureus*, *Staphylococcus xylosus*
Ch1	*Leuconostoc mesenteroides*, Lactiplantibacillus plantarum *, *Streptococcus thermophilus*, *Propionibacterium jensenii*, *Propionibacterium freudenreichii*	*Escherichia coli*, *Citrobacter freundii*, *Enterobacter aerogenes*, *Clostridium tyrobutyricum*, *Listeria monocytogenes*, *Staphylococcus aureus*
Tr1	*Lactococcus lactis*, *Leuconostoc mesenteroides*, Lactiplantibacillus plantarum *, *Lactobacillus acidophilus*, *Propionibacterium jensenii*	*Enterobacter cloacae*, *Citrobacter freundii*, *Bacillus subtilis*, *Clostridium tyrobutyricum*, *Listeria monocytogenes*, *Staphylococcus aureus*
Tr2	*Lactococcus lactis*, *Leuconostoc mesenteroides*, Lactiplantibacillus plantarum *, *Lactobacillus delbrueckii*, *Propionibacterium jensenii*	*Enterobacter aerogenes*, *Enterobacter cloacae*, *Bacillus subtilis*, *Citrobacter freundii*, *Clostridium butyricum*, *Listeria monocytogenes*, *Staphylococcus aureus*, *Staphylococcus xylosus*

* Previous names of species included in the Lactobacillus genus were *Limosilactobacillus fermentum*—*Lactobacillus fermentum*, *Lacticaseibacillus casei*—*Lactobacillus casei*, *Lactiplantibacillus plantarum*—*Lactobacillus plantarum*, *Levilactobacillus brevis*—*Lactobacillus brevis*, *Limosilactobacillus fermentum*—*Lactobacillus fermentum*.

**Table 4:** Counts (log CFU/g) of bacteria in cheeses made from raw cow, ewe, and goat milk.

Cheese Sample	Bacterial Genus/Family						Spores	
	*Lactobacillus*	*Lactococcus*	*Enterobacteriaceae*		*Enterococcus*	*Staphylococcus*	*Bacillus*	*Clostridium*
			*E*. *coli*	Other				
Os1	5.80	6.87	2.71	4.69	3.87	3.43	<2	2.20
	(0.22)	(0.29)	(0.27)	(0.12)	(0.19)	(0.31)	(0.0)	(0.35)
Os2	6.25	7.92	<2	3.32	3.38	2.66	<2	<2
	(0.16)	(0.23)	(0.0)	(0.37)	(0.33)	(0.27)	(0.0)	(0.0)
Os3	5.92	6.73	2.43	<2	5.11	3.52	2.71	<2
	(0.09)	(0.16)	(0.19)	(0.0)	(0.21)	(0.42)	(0.27)	(0.0)
Os4	5.63	6.15	2.23	<2	3.72	2.08	<2	2.38
	(0.11)	(0.28)	(0.21)	(0.0)	(0.22)	(0.36)	(0.0)	(0.28)
Os5	5.94	6.46	<2	3.38	4.62	3.79	<2	3.00
	(0.40)	(0.26)	(0.0)	(0.34)	(0.23)	(0.27)	(0.0)	(0.49)
Os6	7.20	7.73	2.00	<2	4.41	4.08	<2	<2
	(0.10)	(0.09)	(0.36)	(0.0)	(0.17)	(0.24)	(0.0)	(0.0)
Os7	5.97	6.69	2.18	<2	3.58	2.83	<2	<2
	(0.32)	(0.26)	(0.36)	(0.0)	(0.08)	(0.27)	(0.0)	(0.0)
Os8	7.58	8.11	<2	<2	4.52	3.52	<2	<2
	(0.27)	(0.25)	(0.0)	(0.0)	(0.37)	(0.33)	(0.0)	(0.0)
Bu1	7.43	7.81	<2	3.72	4.57	2.94	2.59	<2
	(0.19)	(0.17)	(0.0)	(0.07)	(0.39)	(0.52)	(0.47)	(0.0)
Br1	7.04	7.93	<2	4.34	4.65	2.83	2.63	<2
	(0.25)	(0.19)	(0.0)	(0.08)	(0.35)	(0.29)	(0.24)	(0.0)
Og1	6.72	7.64	<2	2.28	3.61	2.28	<2	<2
	(0.37)	(0.28)	(0.0)	(0.49)	(0.44)	(0.35)	(0.0)	(0.0)
Re1	7.11	8.41	2.45	3.34	4.20	3.96	<2	2.56
	(0.22)	(0.43)	(0.28)	(0.36)	(0.62)	(0.17)	(0.0)	(0.49)
Re2	7.66	8.25	<2	4.88	4.28	3.94	2.11	<2
	(0.31)	(0.58)	(0.0)	(0.41)	(0.24)	(0.37)	(0.46)	(0.0)
Re3	8.11	7.89	<2	5.80	4.87	4.18	<2	<2
	(0.36)	(0.51)	(0.0)	(0.24)	(0.28)	(0.28)	(0.0)	(0.0)
Sg1	5.87	6.68	<2	4.00	3.25	3.20	<2	2.83
	(0.22)	(0.14)	(0.0)	(0.39)	(0.14)	(0.29)	(0.0)	(0.32)
Sg2	6.81	7.15	2.00	2.70	4.00	3.54	3.54	<2
	(0.15)	(0.28)	(0.49)	(0.11)	(0.50)	(0.19)	(0.30)	(0.0)
Se1	5.84	7.25	2.96	6.00	5.75	3.32	<2	<2
	(0.42)	(0.38)	(0.32)	(0.20)	(0.27)	(0.35)	(0.0)	(0.0)
Ch1	5.91	6.58	3.70	2.30	4.89	2.30	2.90	<2
	(0.39)	(0.44)	(0.27)	(0.10)	(0.46)	(0.29)	(0.31)	(0.0)
Tr1	8.46	6.75	<2	2.81	3.04	2.30	<2	2.61
	(0.34)	(0.27)	(0.0)	(0.32)	(0.38)	(0.23)	(0.0)	(0.18)
Tr2	6.04	6.99	<2	<2	4.41	2.15	<2	2.48
	(0.41)	(0.32)	(0.0)	(0.0)	(0.06)	(0.24)	(0.0)	(0.29)

**Table 5:** Distribution of phenotypic and genotypic antibiotic resistance in lactic acid bacteria isolated from regional cheeses produced in Poland.

Species (Number of Isolates)	Number of Antibiotic-Resistant Isolates (%)					
	Phenotypic Resistance ^1^			Genotypic Resistance ^2^		
	TE or E or C	TE + E or TE + C or E + C	TE + E + C	*tet(M,L,W)* or *ermB* or *cat-TC*	*tet(M,L,W) + ermB* or *tet(M,L,W) + cat-TC* or *ermB + cat-TC*	*tet(M,L,W)* + *ermB* + *cat-TC*
*Leuc. lactis* (17)	3 (18)	5 (29)	3 (18)	11 (65)	3 (18)	
*Leuc. mesenteroides* (9)	3 (33)	1 (11)		4 (44)	3 (33)	1 (11)
*Lc. lactis* (11)	3 (36)		1 (10)	5 (45)	1 (10)	
*Lc. garviae* (1)	1 (100)				1 (100)	
*E. faecalis* (1)	1 (100)			1 (100)		
*L. plantarum* (18)	2 (11)	6 (33)	2 (11)	10 (56)	7 (39)	1 (6)
*L. pentosus* (8)	4 (50)	1 (12)	2 (25)	6 (75)	2 (25)	
*L. casei* (5)	2 (40)				2 (40)	1 (20)
*L. paracasei* (4)	1 (25)	1 (25)	1 (25)	2 (50)	2 (50)	
*L. delbrueckii* (3)	2 (67)			2 (67)		
*L. helveticus* (1)				1 (100)		
*L. brevis* (1)						
Total (79)	22 (28)	14 (18)	9 (11)	42 (53)	21 (28)	3 (4)
Total number of resistant isolates	45 (57)			66 (83)		

TE—tetracycline 30 µg, E—erythromycin 15 µg, C—chloramphenicol 30 µg; *tet(M)*, *tet(L)*, *tet(W)*—tetracycline resistance genes, *erm(B)*—erythromycin resistance gene, *cat-TC*—chloramphenicol resistance gene; ^1^ The observed phenotypic resistance of tested strains to only one of the tested antibiotics (TE or E or C), to two antibiotics (TE and E, or TE and C, or E and C), or to all three tested antibiotics (TE, E and C); ^2^ Antibiotic resistance gene(s) present in tested strains, encoding the resistance to only one antibiotic (*tet(M,L,W*) or *ermB* or *cat-TC*), to two antibiotics (*tet(M,L,W*) and *ermB*, or *tet(M,L,W*) and *cat-TC cat-TC*,, or *ermB* and *cat-TC*) or all three tested antibiotics (*tet(M,L,W*) and *ermB* and *cat-TC*).

## Data Availability

Data presented in this study are available on request from the corresponding author.

## Supplementary Materials

The following supporting information can be downloaded at: https://www.mdpi.com/article/10.3390/ani13010168/s1, Table S1: List of reference strains used in this study; Table S2: Genus- and species-specific primers used in the study; Table S3: Antibiotic resistance gene-specific primers used in the study; Table S4: Phenotypic and genotypic resistance of strains isolated from regional cheeses.
