# Supplementary material for: Microbiological Biodiversity of Regional Cow, Goat and Ewe Milk Cheeses Produced in Poland and Antibiotic Resistance of Lactic Acid Bacteria Isolated from Them
Source: Animals (Basel). 2022 Dec 31;13(1):168. doi: 10.3390/ani13010168 (PMC9817895; doi:10.3390/ani13010168)
Supplement: Supplementary file 1 [file animals-13-00168-s001.zip › Table S1 Reference strains.pdf]

Table S1. List of reference strains used in this study.

Strain	Reference Number	Application
Lactobacillus acidophilus	DSM 9126; DSM 20079	DGGE marker, positive control for PCR identification
Lactobacillus amylovorus	DSM 20531	DGGE marker, positive control for PCR identification
Levilactobacillus brevis	DSM 1267; DSM 20054	DGGE marker, positive control for PCR identification
Levilactobacillus brevis	ATCC 367	DGGE marker, positive control for PCR identification; positive control for *cat*-TC gene
Lacticaseibacillus casei	ATCC 334; DSM 20011	DGGE marker, positive control for PCR identification
Lactobacillus delbrueckii ssp. *bulgaricus*	DSM 20080; DSM 20081	DGGE marker, positive control for PCR identification
Lactobacillus delbrueckii ssp. *delbrueckii*	DSM 20074	DGGE marker, positive control for PCR identification
Lactobacillus delbrueckii ssp. *lactis*	DSM 20072	DGGE marker, positive control for PCR identification
Limosilactobacillus fermentum	DSM 20052; DSM 20391	DGGE marker, positive control for PCR identification
Lactobacillus gasseri	ATCC 19992	DGGE marker, positive control for PCR identification
Lactobacillus helveticus	DSM 20075	DGGE marker, positive control for PCR identification
Lentilactobacillus hilgardii	DSM 20176	DGGE marker, positive control for PCR identification
Lacticaseibacillus paracasei	DSM 2649; DSM 5622	DGGE marker, positive control for PCR identification
Lactiplantibacillus paraplantarum	DSM 10667	DGGE marker, positive control for PCR identification
Lactiplantibacillus pentosus	DSM 20314	DGGE marker, positive control for PCR identification
Lactiplantibacillus plantarum	ATCC 8014; DSM 20174	DGGE marker, positive control for PCR identification
Lacticaseibacillus rhamnosus	ATCC 7469; ATCC 9595	DGGE marker, positive control for PCR identification
Limosilactobacillus reuteri	ATCC 55730	DGGE marker; positive control for *tet*W
Lactococcus plantarum	DSM 20686; DSM 20687	DGGE marker, positive control for PCR identification
Lactococcus lactis ssp. *cremoris*	DSM 4367; DSM 20069	DGGE marker, positive control for PCR identification
Lactococcus lactis ssp**.** *lactis*	DSM 4366; DSM 20481	DGGE marker, positive control for PCR identification
Lactococcus raffinolactis	DSM 20443	DGGE marker
Leuconostoc lactis	DSM 8581	DGGE marker, positive control for PCR identification

Leuconostoc mesenteroides ssp. *cremoris*	DSM 20200; DSM 20346	DGGE marker, positive control for PCR identification
Leuconostoc mesenteroides ssp. *dextranicum*	DSM 20484	DGGE marker
Leuconostoc mesenteroides ssp. *mesenteroides*	DSM 20240; DSM 20343	DGGE marker, positive control for PCR identification
Leuconostoc pseudomesenteroides	DSM 5624; DSM 20193	DGGE marker
Streptococcus thermophilus	ATCC 19258	DGGE marker
Propionibacterium acidipropionici	DSM 4900	DGGE marker
Propionibacterium freudenreichii ssp. *freudenreichii*	DSM 20271	DGGE marker
Propionibacterium freudenreichii ssp. *shermanii*	DSM 4902	DGGE marker
Propionibacterium jensenii	DSM 20535	DGGE marker
Propionibacterium thoenii	DSM 20276	DGGE marker
Citrobacter freundii	ATCC 8090	DGGE marker
Enterobacter aerogenes	ATCC 13048	DGGE marker
Enterobacter cloacae	ATCC 13047	DGGE marker
Escherichia coli	ATCC 8739; ATCC 11775	DGGE marker
Escherichia coli	ATCC 25922	DGGE marker, positive control for phenotypic antibiotic resistance
Enterococcus casselliflavus	ATCC 25788	DGGE marker
Enterococcus durans	ATCC 6056	DGGE marker
Enterococcus faecalis	ATCC 29212	DGGE marker; positive control for PCR identification; positive control for phenotypic antibiotic resistance
Enterococcus faecalis	ATCC 51299	DGGE marker, positive control for PCR identification
Enterococcus faecalis	16632EK	Positive control for *tet*W gene
Enterococcus faecium	ATCC 775	DGGE marker, positive control for PCR identification
Enterococcus faecium	ATCC 7171	DGGE marker, positive control for PCR identification
Enterococcus faecium	15555EK	Positive control for *tet*L, *tet*M, *erm*B genes
Enterococcus gallinarum	ATCC 49573	DGGE marker
Enterococcus hire	ATCC 8043	DGGE marker
Listeria monocytogenes	ATCC BAA-751	DGGE marker
Pseudomonas aeruginosa	ATCC 9027	DGGE marker
Staphylococcus aureus	ATCC 43300	DGGE marker
Staphylococcus aureus	ATCC 25923	DGGE marker; positive control for phenotypic antibiotic resistance
Staphylococcus epidermidis	ATCC 49461	DGGE marker
Staphylococcus saprophyticus	ATCC 49453	DGGE marker
Staphylococcus xylosus	ATCC 29971	DGGE marker

Bacillus cereus	ATCC 14579	DGGE marker
Bacillus coagulans	ATCC 7050	DGGE marker
Bacillus subtilis	ATCC 6051	DGGE marker
Clostridium butyricum	ATCC 10702	DGGE marker
Clostridium perfringens	ATCC 13124	DGGE marker
Clostridium sporogenes	ATCC 3584	DGGE marker
Clostridium tyrobutyriucum	ATCC 2637	DGGE marker
