# Supplementary material for: Microbiological Biodiversity of Regional Cow, Goat and Ewe Milk Cheeses Produced in Poland and Antibiotic Resistance of Lactic Acid Bacteria Isolated from Them
Source: Animals (Basel). 2022 Dec 31;13(1):168. doi: 10.3390/ani13010168 (PMC9817895; doi:10.3390/ani13010168)
Supplement: Supplementary file 1 [file animals-13-00168-s001.zip › Table S2 Genus species specific primers.pdf]

Table S2. Genus- and species-specific primers used in the study.

Target	Name	Sequence (5'– 3')	Ta (°C)	Amplicon (bp)	Ref.
Lactobacillus	LbLMA1-rev<br>R16-1	ctcaaaactaaacaaagtttc<br>cttgtacacaccgcccgtca	55	250	[15]
L. casei	Y2<br>casei	cccactgctgcctcccgtaggaagt<br>tgcactgagattcgacttaa	45	290	[16]
L. paracasei	Y2<br>para	cccactgctgcctcccgtaggaagt<br>caccgagattcaacatgg	45	220	
L. rhamnosus	Y2<br>rham	cccactgctgcctcccgtaggaagt<br>tgcatcttgatttaattttg	45	220	
L. brevis	Bre-16S.L<br>Bre-ITS.R	gtgagataaccttcgggagt<br>ggtcacttcgtgatcgtcaa	62	316	[17]<br>[18]
L. helveticus	Hel I<br>Hel II	gaagtcatggagagtagagata<br>ctcttctcggtcgccttg	62	178	
L. acidophilus	23-10C<br>Laci-1	cctttccctcacggtactg<br>tgcaaagtggtagcgtaagc	68	210	[19]
L. plantarum	Lpla-3<br>Lpla-2	attcatagtctagttggaggt<br>cctgaactgagagaatttga	60	248	
L. fermentum	Lfer-3<br>Lfer-4	actaacttgactgatctacga<br>ttcactgctcaagtaatcatc	60	192	
L. delbrueckii	20A<br>23B	aattccgtcaactcctcatc<br>tgatccgctgcttcatttca	62	715	[20]
L. delbrueckii ssp. *bulgaricus*	34/2<br>37/1	cgtcaactcctcatcaaccggggct<br>cgccgcccgggtgaaggtg	62	678	
L. pentosus	16<br>Lpe-16	gctggatcacctcctttc<br>atgaaactattaaattggtac	53	220	[21]
Lactococcus	1RL<br>2RR	tttgagagtttgatcctgg<br>tctacgcatttcaccgcta	45	680	[22]
Lc. lactis ssp. *lactis*	LacF<br>LacreR	gtacttgtaccgactggat<br>gggatcatctttgagtgat	58	163	
Lc. lactis ssp. *cremoris*	CreF<br>LacreR	gtgcttgcaccgatttgaa<br>gggatcatctttgagtgat	58	163	
Leuconostoc	LeucA<br>LeucS	cactttgtctccgaagag<br>aagcactgttgtatggga	45	613	[24]
Leu. lactis	Llac-f<br>Llac-r	aggcggcttactggacaac<br>cttagacggctccttccat	60	742	[23,24]
Leu. mesenteroides	Lmes-f<br>Lmes-r	aacttagtgtcgcatgac<br>agtcgagttacagactacaa	60	1150	
Enterococcus	En I<br>En II	tactgaacaaaccattcatgatg<br>aacttcgtcaccaacgcgaac	55	112	[26]
Enterococcus faecium	EfaecF<br>EfaecR	tcaagtacagttagtctttattag<br>acgattcaaagctaactgaatcagt	54	680	[25]
Enterococcus faecalis	EfecF<br>EfecR	ttgaggcagaccagattgacg<br>tatgacagcgactccgattcc	54	941	

Ta – temperature of annealing
