# Supplementary material for: Microbiological Biodiversity of Regional Cow, Goat and Ewe Milk Cheeses Produced in Poland and Antibiotic Resistance of Lactic Acid Bacteria Isolated from Them
Source: Animals (Basel). 2022 Dec 31;13(1):168. doi: 10.3390/ani13010168 (PMC9817895; doi:10.3390/ani13010168)
Supplement: Supplementary file 1 [file animals-13-00168-s001.zip › Table S3 Antibiotic resistance genes.pdf]

Table S3. Antibiotic resistance gene-specific primers used in the study.

Gene*	Primer's name	Sequence (5' – 3')	T_a (°C)	Amplicon (bp)	Ref.
tet(M)	tetMf	gtggacaaaggtacaacgag	62	406	[29]
	tetMr	cggtaaagttcgtcacacac			
tet(L)	tetLf	tggtggaatgatagcccatt	62	229	[30]
	tetLr	caggaatgacagcacgctaa			
tet(W)	tetWf	gagagcctgctatatgccagc	64	168	[31]
	tetWr	gggcgtatccacaatgttaac			
erm(B)	ermBf	catttaacgacgaaactggc	55	425	[32]
	ermBr	ggaacatctgtggtatggcg			
cat-TC	cat-TCf	caatagcgacggagagttagg	55	384	[33]
	cat-TCr	aatcctgcatgataaccatcac			

* tet(M), tet(L), tet(W) – tetracycline resistant genes, erm(B) – erythromycin, cat-TC – chloramphenicol.

Ta – temperature of annealing.
