# Supplementary material for: Microbiological Biodiversity of Regional Cow, Goat and Ewe Milk Cheeses Produced in Poland and Antibiotic Resistance of Lactic Acid Bacteria Isolated from Them
Source: Animals (Basel). 2022 Dec 31;13(1):168. doi: 10.3390/ani13010168 (PMC9817895; doi:10.3390/ani13010168)
Supplement: Supplementary file 1 [file animals-13-00168-s001.zip › Table S4 LAB isolates Antibiotic resistance.pdf]

**Table S4**. Phenotypic and genotypic resistance of strains isolated from regional cheeses

| Cheese | Isolate symbol | Species identified by PCR | Phenotypic antibiotic resistance[1] | Genotypic antibiotic resistance[2] |
|---|---|---|---|---|
| Os1 | M9 | *Lactiplantibacillus pentosus* | TE | *cat-TC* |
| | M18 | *Lactiplantibacillus pentosus* | TE, C, E | *cat-TC* |
| | M22 | *Lactobacillus delbrueckii* | C | - |
| | M27 | *Lactiplantibacillus plantarum* | TE, C, E | *cat-TC* |
| Os2 | M28 | *Lactiplantibacillus pentosus* | - | *cat-TC* |
| | M30 | *Lactiplantibacillus plantarum* | E, C | *erm(B), cat-TC* |
| | M39 | *Lactiplantibacillus pentosus* | TE, C, E | *cat-TC* |
| | M41 | *Lactiplantibacillus plantarum* | - | *erm(B), cat-TC* |
| Os3 | M42 | *Lactiplantibacillus plantarum* | - | - |
| | M45 | *Lactiplantibacillus pentosus* | TE | *cat-TC* |
| | M46 | *Lactiplantibacillus plantarum* | TE, C | *erm(B), cat-TC* |
| | M47 | *Lactiplantibacillus pentosus* | TE, C | *erm(B), cat-TC* |
| Os4 | BFM 7a | *Leuconostoc lactis* | E | *tet(M)* |
| | BFM 9b | *Leuconostoc lactis* | E | *erm(B)* |
| | BFM 13 | *Leuconostoc mesenteroides* | - | *tet(M), erm(B)* |
| Os5 | BFM 15a | *Leuconostoc lactis* | TE, E | *tet(M)* |
| | BFM 16b | *Leuconostoc mesenteroides* | TE | *tet(M)* |
| | BFM 19 | *Leuconostoc mesenteroides* | TE | *tet(M)* |
| Os6 | BFM 20 | *Leuconostoc lactis* | - | *tet(M), erm(B)* |
| | BFM 22 | *Lactococcus lactis* | TE, C, E | *tet(M)* |
| | BFM 24d | *Lacticaseibacillus casei* | - | - |
| Os7 | BFM 25b | *Leuconostoc mesenteroides* | TE, E | *tet(M)* |
| | BFM 26d | *Lacticaseibacillus casei* | E | *tet(M), erm(B), cat-TC* |
| | BFM 28b | *Lactiplantibacillus plantarum* | TE, C | *tet(M), erm(B)* |
| Os8 | BFM 29a | *Lactiplantibacillus plantarum* | TE, C | *tet(M), erm(B), cat-TC* |
| | BFM 30b | *Lacticaseibacillus casei* | - | *tet(M), erm(B)* |
| | BFM 32 | *Lacticaseibacillus casei* | - | *tet(M), cat-TC* |
| Bu1 | BFM 38 | *Leuconostoc lactis* | TE, C | *tet(M)* |
| | BFM 42a | *Leuconostoc lactis* | - | *tet(M)* |
| | BFM 49 | *Leuconostoc mesenteroides* | TE | - |
| Br | B2 | *Lactiplantibacillus plantarum* | TE | *erm(B), cat-TC* |
| | B4 | *Lactiplantibacillus plantarum* | - | *cat-TC* |
| | B5 | *Lacticaseibacillus paracasei* | - | *erm(B)* |
| | B6 | *Lactiplantibacillus plantarum* | - | *erm(B)* |
| | B7 | *Lacticaseibacillus paracasei* | TE, C | *erm(B)* |
| | B8 | *Lactiplantibacillus plantarum* | E | *cat-TC* |
| Og1 | Lb48 | *Levilactobacillus brevis* | - | - |
| | Lb49 | *Lactobacillus delbrueckii* | TE | *cat-TC* |
| | Lb50 | *Lactiplantibacillus pentosus* | TE | *cat-TC* |
| Re1 | Lb51 | *Lactobacillus helveticus* | - | *erm(B)* |
| | Lb52 | *Lactiplantibacillus plantarum* | TE, C, E | *erm(B), cat-TC* |
| Re2 | Lb54 | *Lactiplantibacillus plantarum* | TE, C | *cat-TC* |
| | Lb55 | *Lactiplantibacillus plantarum* | - | *erm(B)* |
| Re3 | Lb65 | *Lactiplantibacillus pentosus* | E | *erm(B), cat-TC* |
| | Lb70 | *Lactiplantibacillus plantarum* | - | *cat-TC* |
| Sg1 | P11 | *Lactococcus lactis* | - | - |
| | P12 | *Leuconostoc lactis* | - | - |
| | P21 | *Leuconostoc lactis* | - | *tet(W)* |
| | P22 | *Lactococcus garviae* | TE | *tet(L), cat-TC* |
| | P23 | *Lactococcus lactis* | - | *erm(B)* |

| | | | | |
|---|---|---|---|---|
| Sg2 | P71 | *Lactococcus lactis* | - | *tet(W), tet(L), erm(B)* |
| | P72 | *Leuconostoc lactis* | E, C | *erm(B)* |
| | P73 | *Lactococcus lactis* | C | *erm(B), cat-TC* |
| | P74 | *Leuconostoc lactis* | TE, E, C | - |
| | P75 | *Lactococcus lactis* | C | *cat-TC* |
| | P76 | *Lactococcus lactis* | - | - |
| | P77 | *Leuconostoc lactis* | TE, C | *tet(L)* |
| Se1 | P82 | *Lactiplantibacillus plantarum* | - | *erm(B)* |
| | P84 | *Leuconostoc mesenteroides* | - | *tet(M), erm(B), cat-TC* |
| | P912 | *Leuconostoc lactis* | TE | *erm(B)* |
| | P921 | *Lactobacillus paracasei* | TE | *tet(M), cat-TC* |
| | P922 | *Leuconostoc lactis* | TE, E, C | - |
| | P93 | *Leuconostoc lactis* | - | *tet(L), erm(B)* |
| | P94 | *Leuconostoc mesenteroides* | - | *tet(W), tet(L), erm(B)* |
| | P95 | *Lactiplantibacillus plantarum* | TE, C | *tet(L)* |
| | P96 | *Lacticaseibacillus paracasei* | TE, E, C | *tet(L), cat-TC* |
| | P97 | *Leuconostoc lactis* | TE, C | *cat-TC* |
| Ch1 | P51 | *Leuconostoc mesenteroides* | - | *tet(M), erm(B)* |
| | P52 | *Lactococcus lactis* | TE, E, C | *tet(M)* |
| | P53 | *Lacticaseibacillus casei* | TE | - |
| Tr1 | P32 | *Enterococcus faecium* | TE | *cat-TC* |
| | P34 | *Lactococcus lactis* | C | - |
| | P35 | *Lactococcus lactis* | - | *cat-TC* |
| | P42 | *Leuconostoc mesenteroides* | - | *cat-TC* |
| | P43 | *Leuconostoc lactis* | - | *tet(M)* |
| Tr2 | P102 | *Lactiplantibacillus plantarum* | - | *cat-TC* |
| | P105 | *Lactococcus lactis* | - | - |
| | P106 | *Leuconostoc lactis* | TE, E, C | *tet(L), erm(B)* |
| | P107 | *Lactobacillus delbrueckii* | - | *tet(M)* |

Species previously belonging to the *Lactobacillus* genus are: *Limosilactobacillus fermentum – Lactobacillus fermentum*, *Lacticaseibacillus casei – Lactobacillus casei*, *Lacticaseibacillus paracasei – Lactobacillus paracasei*, *Lactiplantibacillus plantarum – Lactobacillus plantarum*, *Lactiplantibacillus pentosus – Lactobacillus pentosus*, *Levilactobacillus brevis – Lactobacillus brevis*, *Limosilactobacillus fermentum – Lactobacillus fermentum*.

[1] TE – tetracycline 30 µg, E – erythromycin 15 µg, C – chloramfenicol 30 µg.

[2] Tetracycline-resistance genes: *tet(M)*, *tet(L)*, *tet(W)*; erythromycin-resistance gene: *erm(B)*; chloramphenicol-resistance gene *cat-TC*.
